# Prior tuberculosis, radiographic lung abnormalities and prevalent diabetes in rural South Africa

**DOI:** 10.1186/s12879-024-09583-8

**Published:** 2024-07-11

**Authors:** Alison C. Castle, Yumna Moosa, Helgard Claassen, Sheela Shenoi, Itai Magodoro, Jennifer Manne-Goehler, Willem Hanekom, Ingrid V. Bassett, Emily B. Wong, Mark J. Siedner

**Affiliations:** 1 Africa Health Research Institute, KwaZulu-Natal, Durban, South Africa; 2 Division of Infectious Diseases, Massachusetts General Hospital, Boston, MA United States of America; 3 Harvard Medical School, Boston, MA United States of America; 4 University of KwaZulu-Natal, KwaZulu-Natal, Durban, South Africa; 5 Division of Infectious Diseases, Yale School of Medicine, New Haven, Connecticut, USA; 6 Department of Medicine, University of Cape Town, Cape Town, South Africa; 7 Division of Infectious Diseases, University of Alabama Birmingham, Birmingham, AL United States of America

**Keywords:** Diabetes, Prior tuberculosis, Chest imaging

## Abstract

**Background:**

Growing evidence suggests that chronic inflammation caused by tuberculosis (TB) may increase the incidence of diabetes. However, the relationship between post-TB pulmonary abnormalities and diabetes has not been well characterized.

**Methods:**

We analyzed data from a cross-sectional study in KwaZulu-Natal, South Africa, of people 15 years and older who underwent chest X-ray and diabetes screening with hemoglobin A1c testing. The analytic sample was restricted to persons with prior TB, defined by either (1) a self-reported history of TB treatment, (2) radiologist-confirmed prior TB on chest radiography, and (3) a negative sputum culture and GeneXpert. Chest X-rays of all participants were evaluated by the study radiologist to determine the presence of TB lung abnormalities. To assess the relationships between our outcome of interest, prevalent diabetes (HBA1c ≥6.5%), and our exposure of interest, chest X-ray abnormalities, we fitted logistic regression models adjusted for potential clinical and demographic confounders. In secondary analyses, we used the computer-aided detection system CAD4TB, which scores X-rays from 10 to 100 for detection of TB disease, as our exposure interest, and repeated analyses with a comparator group that had no history of TB disease.

**Results:**

In the analytic cohort of people with prior TB (*n* = 3,276), approximately two-thirds (64.9%) were women, and the average age was 50.8 years (SD 17.4). The prevalence of diabetes was 10.9%, and 53.0% of people were living with HIV. In univariate analyses, there was no association between diabetes prevalence and radiologist chest X-ray abnormalities (OR 1.23, 95%CI 0.95–1.58). In multivariate analyses, the presence of pulmonary abnormalities was associated with an 29% reduction in the odds of prevalent diabetes (aOR 0.71, 95%CI 0.53–0.97, *p* = 0.030). A similar inverse relationship was observed for diabetes with each 10-unit increase in the CAD4TB chest X-ray scores among people with prior TB (aOR 0.92, 95%CI 0.87–0.97; *p* = 0.002), but this relationship was less pronounced in the no TB comparator group (aOR 0.96, 95%CI 0.94–0.99).

**Conclusions:**

Among people with prior TB, pulmonary abnormalities on digital chest X-ray are inversely associated with prevalent diabetes. The severity of radiographic post-TB lung disease does not appear to be a determinant of diabetes in this South African population.

## Introduction

Tuberculosis (TB) affects 10 million people annually and is one of the top ten causes of death worldwide [1]. In low- and middle-income countries where the TB burden remains high, the incidence of diabetes is expected to double by 2045 [2]. This intersection of epidemics poses a major public health concern because type 2 diabetes not only increases the risk of developing active TB disease but also worsens TB outcomes [3, 4]. The complex interplay between TB and diabetes is estimated to be responsible for 15% of TB cases worldwide, a figure that might increase above 35% in the next decade due to the expanding diabetes epidemic [5, 6]. 

The association between diabetes and TB is increasingly recognized as bidirectional, with evidence suggesting that TB independently increases the incidence of diabetes [7–10]. Chronic inflammation, a known consequence of active TB, is implicated in the development of metabolic syndrome, contributing to insulin resistance [11]. TB has been shown to modulate adipose tissue function via inflammatory immune responses that are linked to early type 2 diabetes pathogenesis [12–14]. In studies of individuals with active TB disease and latent infection, TB has been shown to increase the risk of hyperglycemia and type 2 diabetes [7, 8, 15, 16]. These data suggest that while inflammation during TB has protective effects on *Mycobacterium tuberculosis*, it could also increase susceptibility to diseases such as type 2 diabetes over time.

However, data on the population effect of TB on diabetes risk are sparse [8]. Although transient hyperglycemia during active TB typically resolves with completion of treatment [17, 18], the long-term consequences of this immune response remain uncertain. This phenomenon may be akin to gestational diabetes, where women have resolution of glucose intolerance after delivery, but their risk for metabolic syndrome and insulin resistance later in life is nearly seven times greater than that of women without gestational diabetes [19]. Findings from our prior work in a rural South African population highlight a potential link between prior TB disease, HIV, and diabetes [20]. Specifically, HIV-negative men who completed TB treatment had a greater prevalence of diabetes than their TB-free counterparts [20]. 

In this analysis, we aimed to investigate whether the severity of resolved TB may be associated with the prevalence of diabetes. Current theories suggest that the immune response to TB not only dictates the extent of radiographic lung damage but also intersects with the pathways leading to insulin resistance and type 2 diabetes [14, 21–23]. Chest X-ray lung abnormalities are a surrogate measurement of both the severity of TB disease and the host immune response [24–26]. Therefore, we hypothesize that destructive lung abnormalities on chest radiography, indicative of a robust immunological response, may be associated with a greater prevalence of diabetes. In this study, we leveraged data from a cross-sectional, thoroughly characterized population cohort in rural KwaZulu-Natal to evaluate the association between diabetes and chest X-ray abnormalities among individuals with a history of TB.

## Methods

### Study population and procedures

We analyzed data from persons aged 15 years or older who participated in the Vukuzazi Study. As previously reported, the Vukuzazi Study was a population-wide, cross-sectional study that enrolled residents in the uMkhanyakude District of KwaZulu-Natal, South Africa [27]. All residents of the southern portion of the Africa Health Research Institute Health and Demographic Surveillance System (AHRI HDSS) were visited at home and invited to participate in a mobile health screening that involved traveling through the study catchment area between May 2018 and March 2020. The population in this region consists largely of individuals of black African descent, and there are high rates of poverty, with more than half of the adults unemployed and only two-thirds having access to piped water in their homes [28]. Participants completed questionnaires on sociodemographic information; smoking status; alcohol use; medications; and medical history, including TB, diabetes, and HIV diagnosis and treatment. Anthropometrics were measured by study-enrolled nurses and obtained according to the WHO STEPwise Approach to NCD Risk Factor Surveillance protocol [29]. 

All nonpregnant participants were screened for TB by digital chest X-ray. Because it was not feasible for the study radiologist to review chest X-rays in real time at mobile health camps, we used CAD4TB version 5 software to identify lung field abnormalities to determine eligibility for sputum collection (Delft Imaging Systems, Hertogenbosch, Netherlands) [30, 31]. Sputum was collected from participants who either reported at least one TB symptom (cough, fever, weight loss, night sweats) or had abnormal lung fields according to a predetermined CAD4TB threshold of 25 [30]. 

Following the study visit, an experienced radiologist, blinded to the CAD4TB participant data, evaluated each chest X-ray. The participants who were determined to have abnormal lung fields by the radiologist but whose CAD4TB scores were below the triage threshold of 25 were revisited by the research team for sputum collection. Chest X-rays were also assessed for the presence of specific lung field abnormalities, including cavitation, fibrosis, calcification, pleural thickening, consolidation, nodules, reticular markings, masses, or other abnormalities. CAD4TB version 6 was applied retrospectively and was used in this analysis. The CAD4TB employs deep learning algorithms to automatically detect TB from chest X-ray images. The output from the algorithm is a heatmap indicating suspicious areas in the lungs and an overall score ranging from 10 to 100, with a higher score indicating a greater likelihood of TB based on the analysis.

Sputum samples were tested for *Mycobacterium tuberculosis* by the Xpert^®^ MTB/RIF Ultra test (Cepheid, Sunnyvale, USA) and liquid mycobacterial culture (BACTEC™ MGIT™ 960 System, Becton Dickinson, Berkshire, UK). Nonfasting venipuncture whole-blood samples were collected for hemoglobin A1c (VARIANT II TURBO hemoglobin test system (Bio-Rad, Marnes-la-Coquette, France)) and HIV (Genscreen Ultra HIV Ag-Ab enzyme immunoassay (Bio-Rad)). Participants with a positive HIV immunoassay had a reflex HIV-1 RNA viral load measured (Abbott RealTime HIV-1 Viral Load, Abbott, Illinois, USA).

### Study definitions

To test our hypothesis that the severity of prior TB pulmonary abnormalities is associated with greater diabetes prevalence, we restricted the cohort to participants with prior TB based on either a self-reported history of at least one course of TB treatment or chest radiography interpreted by the study radiologist as “definitive old TB”. Participants with active TB, defined as a positive Xpert MTB/RIF, a positive *Mycobacterium tuberculosis* culture, or who were currently receiving TB therapy at the time of study enrollment, were excluded from the primary analysis because active TB disease may result in stress hyperglycemia [32], and our work focused on diabetes following TB disease. We also excluded participants with active cancer or autoimmune diseases and those who reported taking antiepileptic or glucocorticoid medications because these conditions and therapies can affect hemoglobin A1c.

Our primary exposure of interest was the presence of lung abnormalities, as determined by the study radiologist. The reported abnormalities included cavitation, fibrosis, calcification, pleural thickening, consolidation, nodules, reticular markings, masses, and other abnormalities. Our primary outcome of interest was diabetes, defined based on current World Health Organization diagnostic thresholds as a hemoglobin A1c ≥6.5% (48.0 mmol/mol) [33]. Participants who self-reported a diagnosis of diabetes and reported use of medication within the last two weeks were also considered to have diabetes, irrespective of their current hemoglobin A1c.

We considered age, sex, smoking status, alcohol use status, waist circumference, HIV serostatus, and socioeconomic status as potential confounders of the relationship between pulmonary abnormalities and diabetes in regression models. Participants who reported current or past smoking history were characterized as ever smokers, and those who reported drinking alcohol in the last 12 months were characterized as consuming alcohol. Participants with a positive HIV ELISA were defined as having HIV. Controlled disease was defined as HIV positive on treatment and viral load < 40 copies/uL. Waist circumference, measured in centimeters, was selected as the primary anthropometric measure used in our regression analyses because waist circumference has greater validity for identifying diabetes than other measures [34]. Socioeconomic status was estimated using household asset ownership data collected within two years of the Vukuzazi enrollment date to generate a relative wealth index, divided into quintiles, as developed by Filmer and Pritchett [35, 36].

In sensitivity analyses, we reclassified the exposure of interest as the degree of lung abnormalities on chest radiography, quantified as a continuous measure by the computer-aided detection system CAD4TB version 6. CAD4TB was developed to detect persons with acute TB disease, but its ability to distinguish between those with prior TB and those with active TB disease is poor [37–39]. Many individuals with previously treated TB have sequelae of persistent lung field abnormalities; therefore, we used the CAD4TB output to reflect lung parenchymal abnormalities among individuals with prior TB. Additionally, we evaluated the relationship between pulmonary abnormalities and diabetes in a non-TB comparator group. Those without a history of TB consisted of participants who reported never receiving TB treatment or who did not have radiographic evidence of probable or definitive TB on chest imaging as determined by the radiologist.

### Statistical methods

We first summarized participant characteristics, stratified based on the presence or absence of pulmonary abnormalities detected by the study radiologist. Histograms of CAD4TB scores and radiologist abnormalities were generated to compare differences among persons with prior TB stratified by diabetes disease status.

We fitted logistic regression models with diabetes as the dependent variable and radiologist characteristics of abnormal lung findings as the independent variable to determine the unadjusted relationship between pulmonary abnormalities and odds of diabetes among persons with prior TB. We then fitted multivariable logistic regression models adjusted for known clinical and demographic confounders. In sensitivity analyses, we fitted logistic regression models using CAD4TB scores (every 10 units) as the exposure variable. Finally, we assessed the relationship between CAD4TB score and diabetes prevalence among the non-TB comparator group. All sensitivity analyses were adjusted for the potential clinical and demographic confounders listed above. Statistical analyses were conducted in Stata (version 17, StataCorp, College Station, Texas, USA).

## Results

**Fig. 1:**
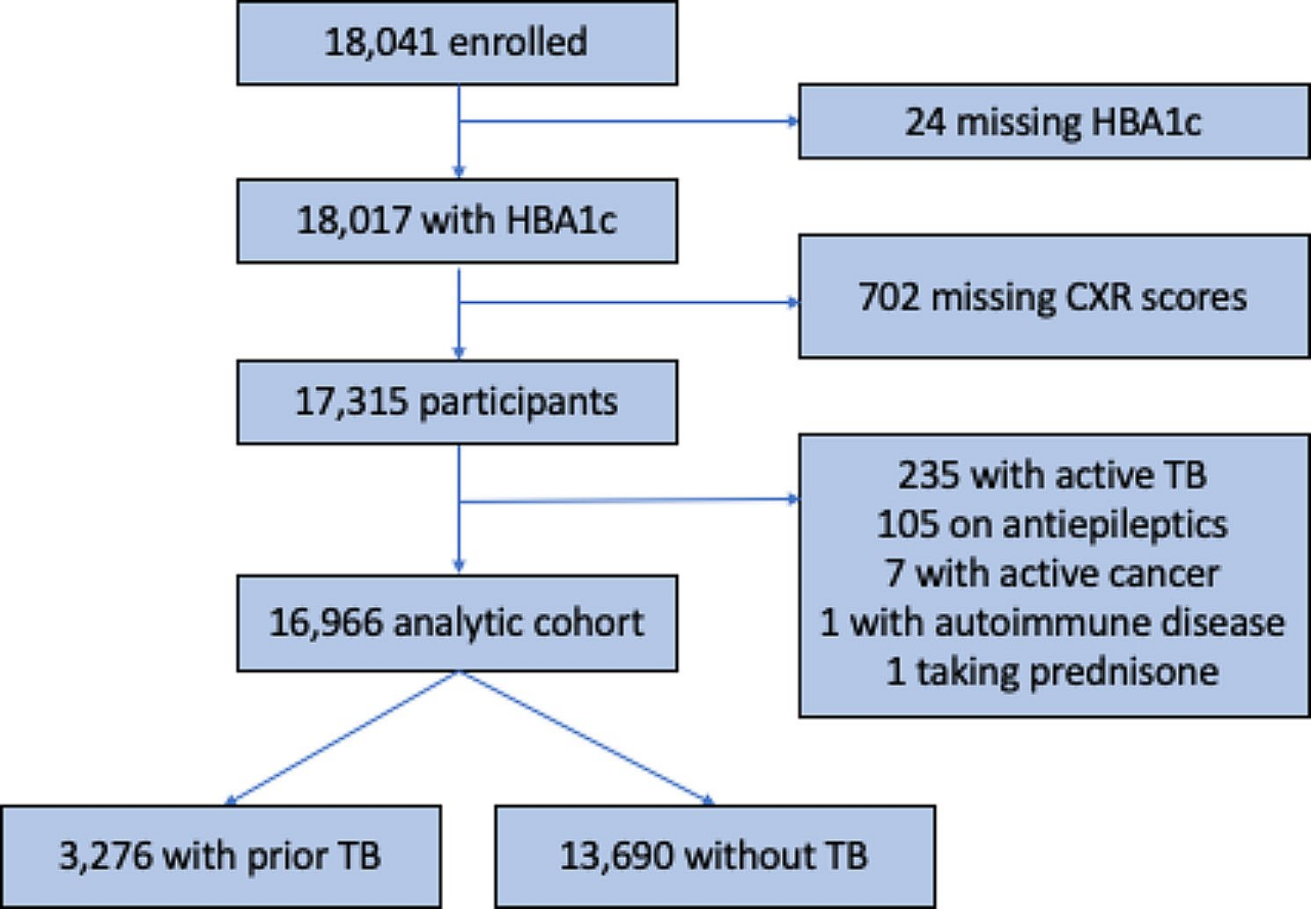
Primary analytic cohort flowchart

Of the eligible participants within the catchment area (*n* = 34,721), 18,041 (51.9%) were enrolled in the Vukuzazi study. Among the enrolled participants, 17,315 (96.0%) had both radiographic and hemoglobin A1c data available. Of these participants, we excluded 235 (1.4%) with active TB, 105 (0.6%) taking antiepileptics, 7 (< 0.1%) with active cancer, 1 (< 0.1%) with autoimmune disease, and 1 (< 0.1%) taking prednisone, resulting in 16,966 individuals (Fig. 1). The analytic sample for our primary analysis consisted of 3,276 participants who met the criteria for prior TB. The non-TB comparison group consisted of 13,960 participants who did not have prior TB treatment nor suspected TB on imaging (Fig. 1).

The mean age of the participants in the prior TB group was 50.8 years (SD 17.4), and 64.9% of the participants were women (Table 1). Participants with pulmonary abnormalities were significantly older than those without radiographic abnormalities (54.0 years (SD 17.5) versus 42.5 years (SD 14.1), *p* < 0.001) Additionally, the proportion of women was lower among those with pulmonary abnormalities compared to those without (63.0% versus 69.8%, *p* < 0.001). Smoking and alcohol consumption rates were similar between the two groups (smoking 13.0% versus 11.4%; alcohol consumption 15.9% versus 17.5%). HIV serostatus differed significantly between the two groups. Among participants with pulmonary abnormalities, 43.6% were living with HIV, compared to 77.0% in the group without radiographic abnormalities. There were no significant differences in HIV disease control or time elapsed since TB diagnosis between the two groups. The prevalence of diabetes was higher in the group with TB pulmonary abnormalities (11.4%) compared to those without radiographic abnormalities (9.5%), however this was not statistically significant. (Table 1).

**Table 1:** Characteristics of persons with prior TB stratified by radiologist determined pulmonary abnormalities

Characteristic	Total (*N* = 3,276)	Presence of TB pulmonary abnormalities (*N* = 2,352)	Absence of TB pulmonary abnormalities (*N* = 924)	*p*-value^
Female (%)	2,126 (64.9)	1,481 (63.0)	645 (69.8)	< 0.001
Age (years)	50.8 (17.4)	54.0 (17.5)	42.5 (14.1)	< 0.001
Socioeconomic status				
Lowest	464 (14.6)	350 (15.3)	114 (12.8)	0.13
Low	901 (28.3)	633 (27.6)	268 (30.2)	
Middle	759 (23.9)	555 (24.2)	204 (22.9)	
High	537 (16.9)	372 (16.2)	165 (18.6)	
Highest	521 (16.4)	383 (16.7)	138 (15.5)	
Ever smoker	410 (12.5)	305 (13.0)	105 (11.4)	0.212
Alcohol use	535 (16.3)	373 (15.9)	162 (17.5)	0.244
Mean BMI (kg/m2)	26.7 (6.7)	26.4 (6.5)	27.7 (7.1)	< 0.001
Mean Waist Circumference (cm)	87.4 (14.9)	86.7 (14.6)	89.1 (15.4)	< 0.001
Living with HIV	1,736 (53.0)	1,025 (43.6)	711 (77.0)	< 0.001
Controlled HIV*	1,446 (83.3)	849 (82.9)	597 (84.0)	0.561
Time from TB diagnosis (years)	11.3 (9.4)	11.8 (10.7)	10.7 (8.0)	0.169
Completed TB treatment courses				< 0.001
Never	1,412 (43.1)	1,405 (59.7)	7 (0.8)	
Once	1,565 (47.8)	742 (31.6)	823 (89.1)	
2–3 courses	256 (7.8)	179 (7.6)	77 (8.3)	
4–5 TB courses	12 (0.4)	10 (0.4)	2 (0.2)	
>5 TB courses	31 (1.0)	16 (0.7)	15 (1.6)	
Diabetes	357 (10.9)	269 (11.4)	88 (9.5)	0.114

Values are mean (standard deviation) or number (%)

^*p*-values calculated for comparisons between pulmonary abnormality groups by Chi-square (categorical variables) and Kruskal-Wallis (continuous variables)

*Controlled disease defined as HIV positive on treatment and viral load < 40 copies/uL

**Fig. 2:**
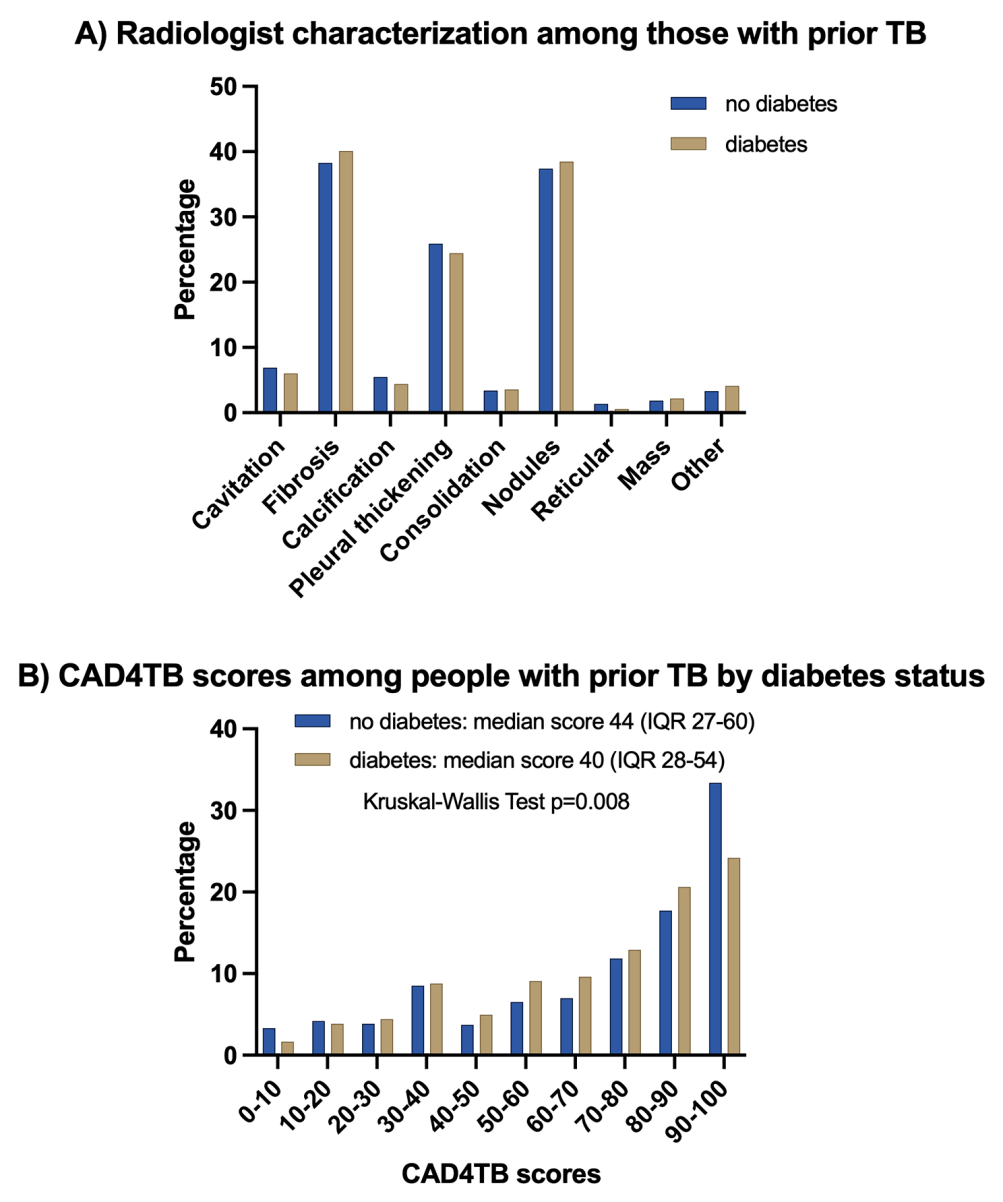
Radiologist Characterization and CAD4TB Score Distribution by Diabetes Status in Individuals with Prior Tuberculosis (TB). Figure 2 A shows the radiologist’s characterization of TB-related abnormalities in chest radiographs, comparing individuals with and without diabetes. Categories include fibrosis, calcification, chronic infiltration, pleural thickening, consolidation, nodules, reticular marks, and other findings. Figure 2B displays the percentage distribution of CAD4TB scores, stratified by diabetes status in patients with a history of TB. Individuals without diabetes had a mean CAD4TB score of 44 (interquartile range, IQR, 27–60), while those with diabetes had a mean score of 40 (IQR 28–54). Statistical analysis using the Kruskal-Wallis Test indicates a significant difference in CAD4TB scores by diabetes status (*p* = 0.008)

In univariate analyses, there was no association between diabetes prevalence and radiologist determined chest X-ray abnormalities among persons with prior TB (OR = 1.23, 95% CI = 0.95–1.58; *p* = 0.114) (Table 2). After adjusting for age, sex, waist circumference, HIV status, smoking, alcohol use, and socioeconomic status, the presence of pulmonary abnormalities was found to be inversely associated with the prevalence of diabetes (aOR 0.71, 95% CI 0.53–0.97; *p* value = 0.030; Table 2). In sensitivity analyses, among the prior TB group, those with diabetes had lower median CAD4TB scores than individuals without diabetes (median score 40 (IQR 27–60) versus 44 (IQR 28–54) among those without diabetes, *p* = 0.008) (Fig. 2). In logistic regression models, CAD4TB scores were also found to be inversely associated with the prevalence of diabetes (aOR 0.92 for each 10-point increase in CAD score, 95% CI 0.87–0.97; *p* value = 0.002; Table 2). In comparison, within the non-TB comparator group, a similar 10-unit increase in CAD4TB score was linked to a 4% reduction in the odds of diabetes (aOR 0.96, 95% CI 0.94–0.99; *p* value = 0.013).

**Table 2:** Logistic regression models for diabetes

Characteristic	Unadjusted Odds Ratio (95%CI)	*p* value	Adjusted Odds Ratio (95% CI)	*p* value
Model: Prior TB cohort with radiologist interpretation (*n* = 3,276)				
Radiologist abnormalities	1.23 (0.95–1.58)	0.114	0.71 (0.53–0.97)	**0.03**
Female sex	2.50 (1.90–3.29)	< 0.001	1.27 (0.93–1.74)	0.133
Age (years)	1.04 (1.03–1.05)	< 0.001	1.03 (1.02–1.04)	**< 0.001**
Waist Circumference (cm)	1.93 (1.74–2.13)	< 0.001	1.61 (1.45–1.79)	**< 0.001**
Living with HIV	0.37 (0.29–0.47)	< 0.001	0.55 (0.42–0.74)	**< 0.001**
Ever smoker	0.16 (0.08–0.32)	< 0.001	0.50 (0.24–1.03)	0.063
Consumes alcohol	0.23 (0.14–0.38)	< 0.001	0.49 (0.28–0.86)	**0.013**
Socioeconomic status	1.10 (1.03–1.16)	0.001	1.07 (1.00-1.13)	**0.035**
**Model: Prior TB cohort with CAD4TB scores (***n* = 3,**276)**				
CAD4TB scores	0.97 (0.94–1.01)	0.21	0.92 (0.87–0.97)	**0.002**
Female sex	2.50 (1.90–3.29)	< 0.001	1.19 (0.87–1.64)	0.283
Age (years)	1.04 (1.03–1.05)	< 0.001	1.03 (1.03–1.04)	**< 0.001**
Waist Circumference (cm)	1.93 (1.74–2.13)	< 0.001	1.57 (1.41–1.76)	**< 0.001**
Living with HIV	0.37 (0.29–0.47)	< 0.001	0.58 (0.44–0.77)	**< 0.001**
Ever smoker	0.16 (0.08–0.32)	< 0.001	0.51 (0.24–1.06)	0.071
Consumes alcohol	0.23 (0.14–0.38)	< 0.001	0.50 (0.29–0.89)	**0.018**
Socioeconomic status	1.10 (1.03–1.16)	0.001	1.06 (1.00-1.13)	0.047
**Model: Non-TB comparator group (***n* = 13,**998)**				
CAD4TB scores	1.11 (1.09–1.13)	< 0.001	0.96 (0.94–0.99)	**0.013**
Female sex	2.40 (2.07–2.78)	< 0.001	0.99 (0.83–1.19)	0.941
Age (years)	1.06 (1.05–1.06)	< 0.001	1.04 (1.04–1.05)	**< 0.001**
Waist Circumference (cm)	2.18 (2.07–2.31)	< 0.001	1.66 (1.56–1.77)	**< 0.001**
Living with HIV	0.54 (0.47–0.62)	< 0.001	0.70 (0.59–0.82)	**< 0.001**
Ever smoker	0.40 (0.29–0.55)	< 0.001	0.98 (0.66–1.44)	0.901
Consumes alcohol	0.26 (0.19–0.35)	< 0.001	0.44 (0.31–0.62)	**< 0.001**
Socioeconomic status	1.11 (1.07–1.14)	< 0.001	1.05 (1.01–1.08)	**0.009**

## Discussion

In a large, well-characterized population in rural South Africa, we found that more severe lung abnormalities on digital chest X-rays were inversely associated with prevalent diabetes. Specifically, for those with a history of prior TB, the presence of radiologist detected pulmonary abnormalities was associated with an 29% reduction in the odds of prevalent diabetes. These results contradict our initial hypotheses that greater pulmonary abnormalities may increase diabetes prevalence due to the proposed aberrant inflammation resulting from prior TB disease [40, 41]. Further analyses using CAD4TB scores of chest X-rays corroborated these findings as lung abnormalities detected by the deep learning technology were also associated with lower odds of prevalent diabetes.

Several studies have shown that individuals with coexisting active TB and diabetes often exhibit atypical chest imaging features, such as lower lung field lesions and cavitations [42–48]. Given this relationship, Geric et al. leveraged computer-aided detection software (CAD) to determine if diabetes is associated with the radiographic presentation of tuberculosis among participants in Pakistan. Individuals with diabetes had higher CAD tuberculosis abnormality scores and were more likely to have cavitations outside of the upper lung zones [49]. Our study, distinct from those focusing on active TB, investigated the link between prior TB—on average diagnosed 11 years previously—and metabolic disease using chest radiography. Multiple studies have linked TB to the risk of diabetes [7, 8, 15, 16]. However, the pathophysiology linking post-TB recovery and diabetes risk is not well understood. Current theories suggest that the immune response to TB not only dictates the extent of radiographic lung damage but also intersects with the pathways leading to insulin resistance and type 2 diabetes [21]. Our hypothesis posited that a greater extent of lung damage, as determined by radiographic abnormalities, would convey a more robust immune response and thus be associated with a higher prevalence of diabetes. In contrast, our data revealed an inverse relationship: severe lung abnormalities in TB survivors—and in non-TB controls—were associated with a reduced prevalence of diabetes.

Our findings imply that severe radiographic abnormalities, which may lead to chronic pulmonary disease, may offset the factors that promote diabetes. For example, other causes of lung abnormalities, such as nontuberculosis mycobacterium infection, malignancy, or chronic obstructive pulmonary disease (COPD), could lead to systemic wasting or cachexia, thereby reducing the risk of metabolic complications [50, 51]. Given the high incidence of conditions such as malignancy and atypical infections among individuals living with HIV – who made up 53% of our study sample – this could be a contributing factor to our results. The ECLIPSE study, which analyzed 2,164 individuals with COPD to understand disease heterogeneity, supports our results by showing that women with COPD diagnosed via chest imaging were less likely to have diabetes compared to their male counterparts [52]. Our study also had a high proportion of female participants (64.9%), which may have contributed to the observed lower diabetes prevalence in those with more severe lung pathology. Moreover, the ECLIPSE study identified that individuals with the nonemphysematous type of COPD had twice the odds of having diabetes compared to those with emphysematous COPD [53]. This distinction is important, as the risk factors for diabetes and chronic pulmonary disease manifestations differ. Although pulmonary TB increases the risk of COPD, often leading to bronchiectasis or emphysematous changes, the differences between COPD subtypes in post-TB patients remain unexplored [54]. Further research is needed to distinguish lung disease types in TB survivors and determine which aspects of pulmonary pathology influence metabolic disease risk.

There are other possible factors that may explain our findings. First, survivors of TB have nearly fourfold greater mortality than healthy matched controls without TB [55]. The cross-sectional nature of our study may be subject to a survival bias, such that those with the most severe prior TB disease and risk for diabetes passed away, diminishing or even reversing the relationship between disease severity and diabetes risk. Alternatively, the effect of prior TB on diabetes risk may not be dependent on the severity of radiographic pulmonary abnormalities but rather on other immune- or adipose-related pathophysiologies. Finally, the CAD4TB algorithm has been specifically designed to detect active TB—not post-TB lung disease—by analyzing lung parenchyma and textural characteristics. Although the interpretation of chest X-rays by a radiologist was the primary analysis, chest imaging alone is not precise enough to detect subtle post-TB lung disease in all individuals nor distinguish it from other etiologies causing radiographic abnormalities [56–58]. 

### Strengths and limitations

The strengths of our study include the use of a large population-based cohort that was thoroughly characterized by hemoglobin A1c levels, HIV status, chest X-ray results, sputum culture results, GeneXpert results, and TB treatment data to identify and differentiate individuals with prior TB. Furthermore, anthropometric measures, socioeconomic data, smoking status, and alcohol use were included in our analyses; these variables could confound the relationship between TB-related lung abnormalities and diabetes. Our study has important limitations. The cross-sectional study design prevents causal inference or directionality between digital chest X-ray scores, pulmonary TB, and diabetes. Furthermore, there may be other confounding variables not included in the analysis, such as a family history of diabetes, physical activity, or other non-steroidal anti-inflammatory medications taken by participants. Our data are susceptible to misclassification, as we assume that lung abnormalities quantified by the study radiologist and CAD4TB scores are a result of TB disease. However, our restriction of our sample to those with prior TB partially helps to mitigate this point. Finally, chest radiography, whether evaluated by the study radiologist or CAD4TB software, cannot distinguish former TB from other processes, such as malignancy or environmental exposures.

## Conclusions

In a rural South African population cohort, we found that the degree of lung abnormalities detected via digital chest X-ray was inversely associated with diabetes prevalence, particularly among individuals with a history of TB. This inverse association challenges our initial hypothesis, suggesting that pulmonary abnormalities may counteract the factors that promote the progression of metabolic disease in this population.

## Data Availability

The data and the data dictionary defining each field can be accessed at https://data.ahri.org/index.php/catalog/1006 via the Africa Health Research Institute Data Repository. Please email RDMServiceDesk@ahri.org. Access can be granted after publication and upon approval of the proposed analyses by the Vukuzazi Steering Committee and completion of a data access agreement.
